# Case report: Occupational poisoning incident from a leak of chloroacetyl chloride in Jinan, Shandong, China

**DOI:** 10.3389/fpubh.2023.1215293

**Published:** 2023-08-01

**Authors:** Lanlan Guo, Xiangxing Zhang, Zhiqiang Zhou, Mengdi Shi, Xiangdong Jian, Laidong Dong

**Affiliations:** ^1^Department of Occupational and Environmental Health, School of Public Health, Cheeloo College of Medicine, Shandong University, Jinan, Shandong, China; ^2^Department of Poisoning and Occupational Diseases, Emergency Medicine, Qilu Hospital, Cheeloo College of Medicine, Shandong University, Jinan, Shandong, China; ^3^Department of Outpatient, Qilu Hospital, Cheeloo College of Medicine, Shandong University, Jinan, Shandong, China

**Keywords:** occupational accident, chloroacetyl chloride, chloroacetic acid, myocardial damage, hydrogen chloride

## Abstract

Chloroacetyl chloride is a potent acylation agent that decomposes violently in water to produce chloroacetic acid and irritant hydrogen chloride. It and its decomposition products are corrosive to the eyes, skin, and respiratory system and can cause multiple organ failure. Herein, we report cases of poisoning by chloroacetyl chloride and its decomposition products in the skin and respiratory system. After exposure, one patient developed vomiting, irritability, coma, hypoxemia, hypotension, acidosis, and hypokalemia. Another patient developed bronchiolitis, pneumonia, and decreased vision. One patient died and two recovered. Chloroacetyl chloride and its decomposition products are corrosive and can damage multiple organs after absorption through the skin and respiratory tract, leading to severe heart failure. Cardiogenic shock may be the primary cause of early mortality.

## 1. Introduction

Chloroacetyl chloride (CAC;CAS number: 79-04-9) is an important intermediate in the synthesis of organic compounds, and has the molecular formula C_2_H_2_Cl_2_O (1). It is a colorless or light-yellow liquid at room temperature with an extremely irritating odor (2) and is widely used in the synthesis of pharmaceuticals, pesticides, and additives (3, 4). CAC is used in the second step of the production of the herbicide acetochlor. In which, it is added to N-(2-ethyl-6-methylphenyl) methylimine (C_10_H_13_N), the product of the first step, to form a tertiary amine, which is used in the third step. Here, we report a case of chloroacetyl chloride waste liquid leakage, in which one patient died of neurological damage, heart failure, respiratory failure, and other causes, whereas the other two survived.

## 2. Case description

At approximately 4 a.m. on February 19, 2023, three individuals were working outdoors at a chemical plant that uses chloroacetic acid, chlorine, and sulfur to produce CAC, which produces an acidic waste liquid containing CAC. The purified CAC is then shipped to another plant to produce acetochlor. Their duty was to discharge liquid waste into a waste barrel through a pipeline. Liquid waste accumulates in the reaction kettle during chloroacetyl chloride production. Because of sleepiness and dim light, they did not carefully check whether the waste barrel was clean and dry; there may have been some standing water in the barrel. Therefore, they discharged the liquid waste directly into the barrel (approximately 1.5 m high and 0.8 m diameter cylindrical barrel). The CAC in the waste liquid reacted violently with water, and a lot of heat and hydrogen chloride were generated. The pressure in the waste liquid barrel rose sharply and the barrel exploded, spraying the liquid with what the patient described as a cloud of acrid white smoke. Three workers wore only N95 masks and had no other protective measures. Patients 1 and 2 were closer to the waste liquid bucket and had direct contact with the sprayed liquid and steam, and the skin contact area was large. Patient 3 was at the back, and only the right side of the face had slight contact. Moreover, the exposed area in patient 1 was much larger than that in patient 2, whose face was the most exposed part of his body. Furthermore, his chest injury was caused by the chemicals on his face flowing down his neck to his chest. However, patient 1’s exposure not only affected his face, it also affected his limbs, whose skin corrosion was more serious than that of his face. After the accident, they washed their faces with water; however, they did not change their contaminated clothes. They contacted the person in charge of the factory who sent them to the local hospital. An ophthalmologist rinsed their eyes and faces with eye washes and sent them to our hospital. After admission, the three patients first removed the contaminated clothing, the contaminated skin and eyeball were washed with 5% sodium bicarbonate solution, and tobramycin and dexamethasone eye drops were applied. The symptoms, disease progression, and outcomes differed among the three patients, as described below. This study was approved by the Ethics Committee of Shandong University Qilu Hospital, and informed consent was obtained from all the patients.

### 2.1. Case 1

**Figure 1:**
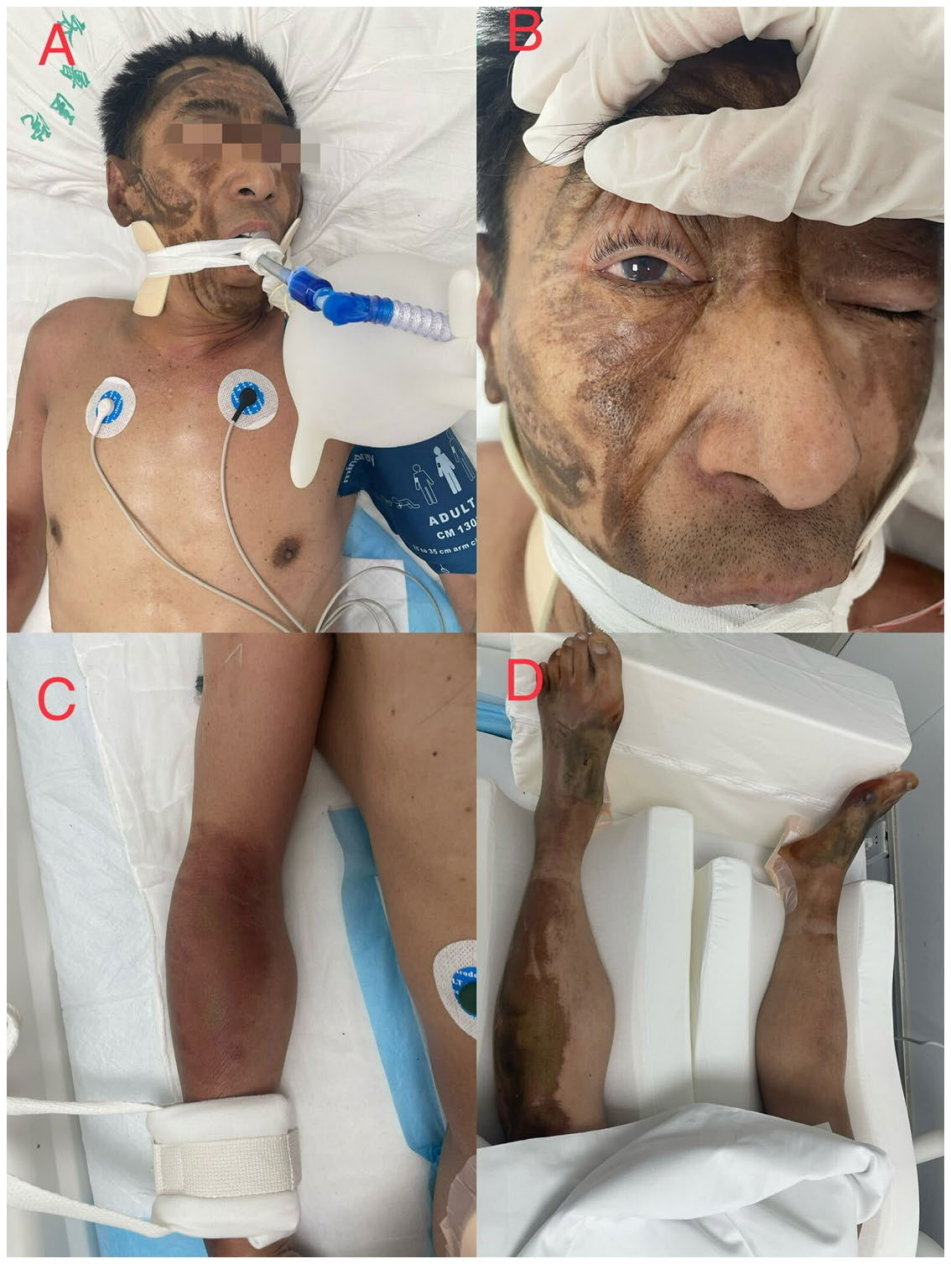
The figure shows burns in patient 1. **(A)** the patient fell into a coma after admission with facial corrosion injury and underwent tracheal intubation, **(B)** blisters are visible in the conjunctiva of the eye, **(C)** and **(D)** chemical burns on the limbs.

Patient 1 had worked in this chemical factory for approximately 8 years and was usually responsible for feeding and receiving waste liquids. We inquired about his past medical history, especially concerning nervous system and cardiopulmonary diseases. His family members reported that he had not suffered from any diseases, taken any medication, or complained of any discomfort at least in the previous year. No abnormality was found in his annual physical examination report. During the accident, the patient dripped on the face and limbs (Figure 1). At approximately 6 o’clock, he vomited, became agitated on the way to our hospital, and lost consciousness at approximately 7 o’clock; oxygen saturation was 60%, and blood pressure (BP) dropped to 70/30 mmHg. The patient was transferred to our hospital at approximately 9 o’clock.

**Figure 2:**
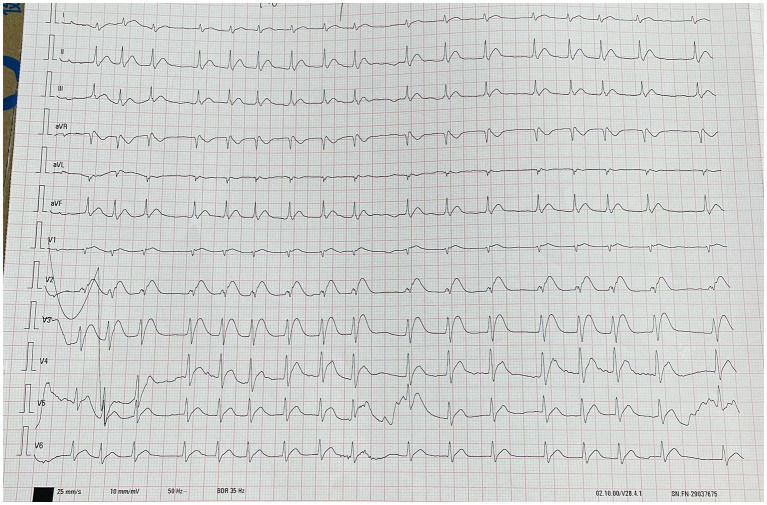
The picture shows the electrocardiogram (ECG) results of the patient about 1 h after admission. This image shows acute anterior wall myocardial damage and degree II atrioventricular block.

The physical examination results of case 1 were as follows: temperature, 36.9°C; heart rate, 75 beats/min; respiratory rate, 20 beats/min; blood pressure, 72/30 mmHg; and oxyhemoglobin saturation, 63%. He was delirious and was unable to cooperate during the physical examination. Flaky brown chemical erosions were observed on the face and limbs, and superficial lymph nodes were small. The pupils were equal and round bilaterally, approximately 3.0 mm in diameter, had a slow reaction to light reflex, and blisters were observed in the bulbar conjunctiva. Cyanosis of the lips and pharyngeal congestion were not observed. The neck was soft and there was no resistance. Chest movements were bilateral, and breath sounds were coarse in both lungs. Dry and wet rales were not observed. The heart sounds were low and dull, and the abdomen was flat without intestinal or peristaltic waves; however, the bowel sounds were normal. There were no deformities of the spine or limbs, physiological reflexes were present, pathological reflexes were not elicited, and the laboratory test results of the patient on the day of admission are shown in Table 1. Blood gas analysis revealed PH of 7.259, serum potassium 3.14 mmol/L, blood sugar 8.0 mmol/L, and lactic acid 2.6 mmol/L. The laboratory test results are presented in Table 1. An electrocardiogram (Figure 2) showed acute anterior myocardial damage and a second-degree atrioventricular block. After admission, the patient was immediately intubated, ventilator-assisted ventilation was performed, and the following drugs were administered: norepinephrine and dopamine were continuously pumped to increase blood pressure; flucloxacillin (1 g, intravenous infusion, every 6 h) to fight infections; dexamethasone (40 mg, intravenous infusion, once a day) to fight inflammation, intoxication, and shock; polyene phosphatidylcholine (232.5 mg, intravenous infusion, once a day) to protect the liver; alanyl glutamine (20 g, intravenous infusion, once a day) and fatty milk amino acid (17%) Glucose (11%) injection to support nutrition; torasemide (20 mg, intravenous injection, twice a day) to promote diuresis; and urinastatin (100,000 units, micropump, every 8 h) to inhibit protease, promote microcirculation, and fight shock. In addition, tobramycin dexamethasone eye drops (one drop in each eye, three times a day), as well as intravenous drip 1,000 mL of normal saline, were administered to replenish fluid and 5% sodium bicarbonate 125 mL was used to correct acidosis. He had a urine volume of less than 50 mL and dark urine 4 h after admission. Approximately 4 h after admission, the patient experienced cardiac arrest and died after ineffective rescue.

**Table 1:** Laboratory test results.

		**Laboratory testing date for 3 patients**							
**Biochemical blood indicators**	**Case 1**	**Case 2**						**Case 3**	**Reference values**
	2.19	2.19	2.21	2.25	3.14	3.17	3.20	2.19	
White blood cells (×10^9^/L)	11.45	12.32	11.39	13.92	9.29	7.26	5.71	11.74	3.5–9.5
Neutrophils (%)	93.78	94.10	79.30	65.60	57.10	53.70	55.20	78.70	40–75
Alanine transaminase (U/L)	36	41	21	33	46	44	53	16	21–72
Aspartate aminotransferase (U/L)	96	47	16	20	19	27	26	24	17–59
Direct bilirubin (μmol/L)	0	0	2.9	3.3	4.3	2.9	2.0	0	0–5
Indirect bilirubin (μmol/L)	10	5	5.0	4.7	7.2	4.3	3.5	33	0–19
Creatine kinase (U/L)	770	286	86	64	57	61	116	40	30–170
Creatine kinase isoenzyme (ng/mL)	23.80	8.30	1.4	1.0	1.1	0.6	0.9	0.50	0–4
Lactate dehydrogenase (U/L)	251	289	194	205	263	276	303	139	120–230
Urea nitrogen (mmol/L)	10.6	7.7	8.20	10.90	7.10	4.80	3.50	8.9	3.2–7.1
Creatinine (μmol/L)	166	59	61	65	78	67	67	68	62–115
Cardiac troponin I (ng/L)	97.27		29.44		2.94	2.44	3.33	3.12	<17.5
Myoglobin (ng/mL)	15312.8								0–70

### 2.2. Case 2

**Figure 3:**
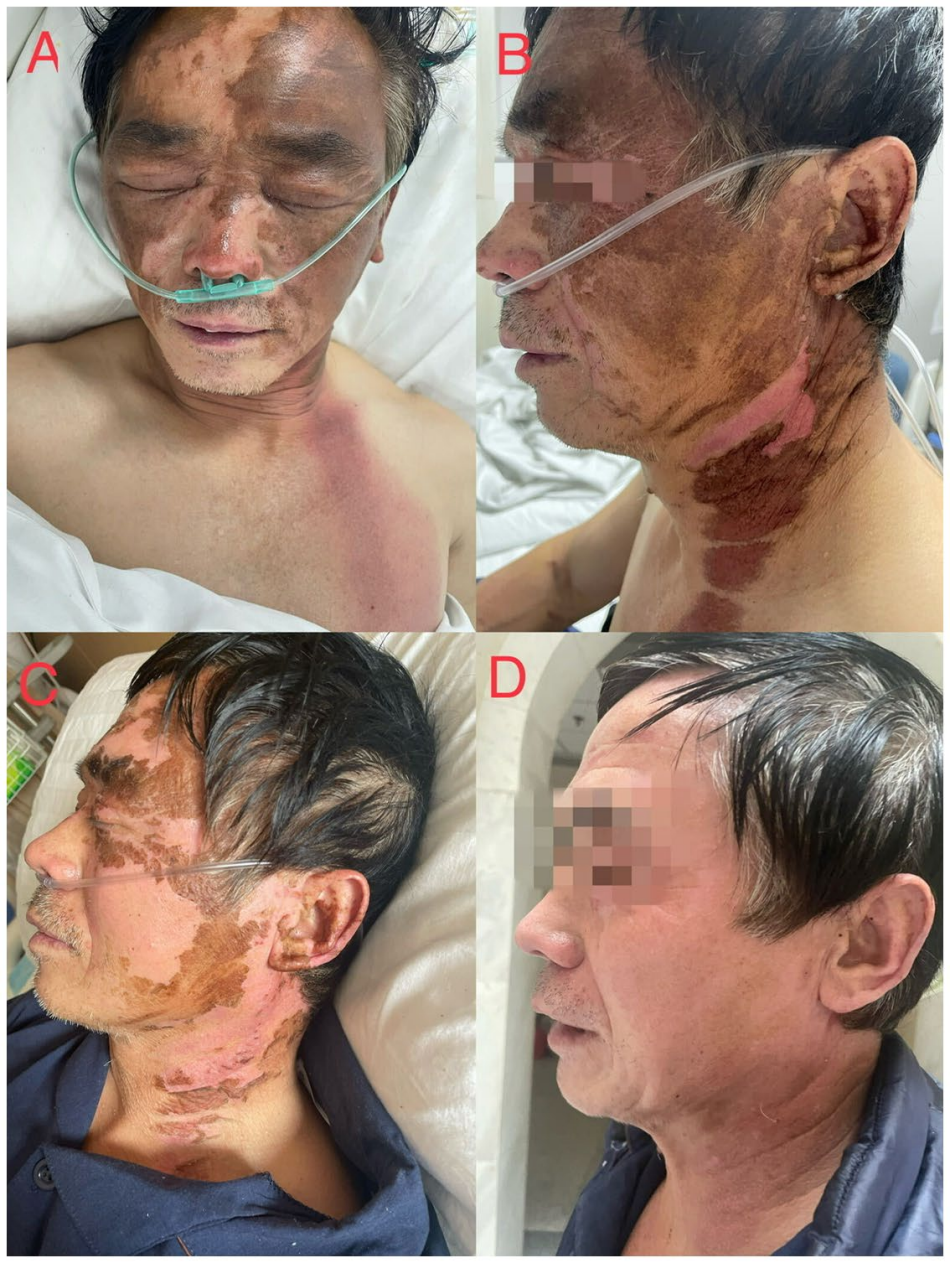
**(A)** Condition of the patient’s facial chemical burns on the day of admission, **(B)** condition of the patient’s face on the fifth day with partial molting, **(C)** condition of the patient’s face on the eighth day with extensive molting, **(D)** condition of the patient’s face on the 24th day with complete molting.

Patient 2 had worked in the factory for more than 10 years and was responsible for feeding and receiving the waste liquid. No cardiopulmonary or other diseases were found during annual physical examinations. In this accident, waste liquid was sprayed onto the face and chest (Figure 3A). At approximately 7 o’clock, he was vomiting and conscious, with stable vital signs, severe conscious pain in the face and eyes, and inability to open eyes. Electrocardiogram monitoring and oxygen inhalation therapy were provided after admission. The patient experienced discomfort behind the sternum and dyspnea. Electrocardiography and chest, brain, and abdominal computed tomography (CT) showed no obvious abnormalities. Simultaneous comprehensive treatments were provided, as follows: flucloxacillin (1 g, intravenous infusion, every 6 h) to fight infections; dexamethasone (40 mg, intravenous infusion, once a day) to fight inflammation, intoxication, and shock; polyene phosphatidylcholine (232.5 mg, intravenous infusion, once a day) to protect the liver; alanyl glutamine (20 g, intravenous infusion, once a day) and fatty milk amino acid (17%) Glucose (11%) injection to support nutrition; torasemide (20 mg, intravenous injection, twice a day) to promote diuresis; urinastatin (100,000 units, micropump, every 8 h) to inhibit protease, promote microcirculation, and fight shock; and tobramycin dexamethasone eye drops (one drop in each eye, three times a day). On Day 2, electrocardiography revealed a prolonged boundary QT interval. On Day 6, chest CT (Figure 3A) showed patchy shadows in the left lower lobe of the lung and a few patchy and ground-glass shadows under the pleura of both lungs, suggesting bronchiolitis in the left lower lobe of the lung. During hospitalization, the patient’s laboratory test indicators (Table 1) and skin corrosion (Figure 3) gradually improved; however, he still had cough and chest tightness. The patient was discharged automatically on the 14th day after the incident and was regularly reviewed. After discharge, the patient’s chest tightness and breathing did not improve, and he experienced blurred vision in his right eye. He returned to our hospital for reexamination on the 22nd day after the accident, and chest CT (Figure 3B) showed new inflammatory and fibrous lesions in the lower lobe of the right lung. Ophthalmic examination showed that the visual acuity of the right eye was 4.3, and that of the left eye was 4.8. The bilateral conjunctival membranes were not congested, the cornea was clear, and small clouds were visible below the cornea of the right eye. The ophthalmologist prescribed sodium hyaluronate eye drops (0.01 mL, three times a day for the affected eye). The patient was readmitted to the hospital for anti-inflammatory and anti-infective treatment and discharged 5 days later. The patient was asked to undergo a regular reexamination. The patient’s pneumonia significantly improved on CT reexamination 2 months after the incident. We asked him to come for regular review. During subsequent telephone follow-up, the patient had no other discomfort except for blurred vision in the right eye.

### 2.3. Case 3

Patient 3 was mainly affected in the right cheek and eye; the right eye was red and swollen with tears and could not open. After admission, electrocardiogram monitoring was performed. The comprehensive treatments for the patient were similar to those of patient 2. The laboratory test results are shown in Table 1. CT of the chest and abdominal brain revealed no abnormalities. The patient could open their eyes without obvious discomfort 3 days later, and he was discharged. During a telephone follow-up and Patient 2’s description in the following month, we learned that Patient 3’s skin chemical burns gradually improved without complaining of significant discomfort.

## 3. Discussion

Chloroacetyl chloride is highly reactive (5) and reacts violently with water to form chloroacetic acid and hydrogen chloride (6). As described by the patient, residual water may have been present in the waste barrel, and CAC was violently hydrolyzed, producing a large amount of heat and steam that caused the barrel to crack, and the waste liquid to spill out, followed by a large area of acrid white smoke.

Occupational exposure to CAC may occur through inhalation or skin contact (7). CAC is corrosive to the skin, with a median lethal dose of 662 mg/kg through percutaneous absorption in rats, 0.1 mL can cause severe eye irritation in rabbits (8), and short-term exposure in humans can cause second or third-degree burns, which are extremely eye-damaging (9). The degradation products of CAC, chloroacetic acid and hydrogen chloride, are considered the main causes of toxicity in this accident. Chloroacetic acid is corrosive and can be rapidly absorbed through the skin, causing metabolic acidosis and multiple organ failure, leading to death (10). Acute high doses of chloroacetic acid cause neurotoxicity and damage to the blood–brain barrier (11, 12), possibly due to oxidative stress damage caused by exposure to peroxides produced by the toxicant, which triggers cell death through mitochondrial dysfunction. This activates the poly-ADP-ribose polymerase and caspase cascades, leading to neuronal apoptosis (10). Patient 1 first developed vomiting and then progressed from extreme agitation to coma, indicating neurological damage. However, neuroimaging could not be performed because of the patient’s critical condition and unstable vital signs.

Animal experiments have shown that the kidneys, brain, thymus, liver, and heart are the target tissues for chloroacetic acid accumulation (13). Accumulation occurs first in myocardial tissue and 4 h later in the cerebellum and liver (14). Chloroacetic acid had the most significant effect on the heart of rats, inhibiting aconitase activity and causing myocardial degeneration, suggesting that large doses of chloroacetic acid could lead to death from heart failure (15). At the same time, CAC can cause myocardial damage (2). Difficult-to-correct hypotension and electrocardiography in patient 1, together with laboratory tests, indicated that cardiogenic shock was the likely immediate cause of death. The borderline electrocardiogram of patient 2 on day 2 and abnormal troponin level (CTNI of 29.44 ng/L) on day 3 indicated transient myocardial damage.

Hydrogen chloride is highly corrosive and can damage the eye and respiratory mucosa even at low concentrations (16). Human inhalation of hydrogen chloride affects the upper and lower respiratory tracts, causing laryngeal edema, bronchial inflammation, alveolar edema, and hypoxemia (17). The respiratory symptoms in patient 2 were prominent. Bronchitis and pneumonia were considered to be caused by the inhalation of hydrogen chloride or acid mixed with chloroacetic acid. Poststernal pain and subpleural inflammation were probably due to chest skin exposure to chloroacetic acid, and the cause of visual acuity loss in patient 2’s right eye was considered to be corrosion of the eyeball by hydrogen chloride and chloroacetic acid. Patient 1 developed hypoxemia approximately 3 h after poisoning. It is believed that hydrogen chloride disrupts the integrity of pulmonary micro vessels, allows fluid and proteins to ooze into the airways and alveoli, inhibits oxygen diffusion, and interferes with surfactants (18).

Symptoms of systemic poisoning with chloroacetic acid usually appear within a few hours, and when they occur, the prognosis is poor (19, 20). Dichloroacetic acid is the drug of choice for MCA poisoning, whereas phenobarbital is usually only used in the absence of DCA and with respiratory and cardiovascular support because of its serious side effects, and N-acetylcysteine can be used as adjuvant therapy (21). Symptomatic treatment, such as actively correcting cerebral edema, metabolic acidosis, and hypokalemia, should be performed after systemic poisoning. Cardiogenic shock due to myocardial damage can be fatal, and early electrocardiogram monitoring and nutritional and myocardial therapies are beneficial to myocardial recovery (22); for renal failure due to rhabdomyolysis, continuous renal replacement therapy (23) or plasma exchange (21) is usually considered. Patient 1 had myoglobinuria and oliguria, which were considered to be due to kidney injury and cardiac under perfusion; however, we did not perform continuous hemofiltration because his blood pressure was difficult to maintain. Patient 2 suggested that chloroacetic acid might cause myocardial damage even in the absence of severe systemic toxic symptoms.

Hydrogen chloride and chloroacetic acid may exert synergistic effects. Hypoxia caused by hydrogen chloride injury to the respiratory system aggravates the nervous system and causes myocardial damage. Chloroacetic acid damage to the central nervous system also inhibits respiration and affects cardiovascular function, leading to severe hypoxemia and hypotension, as in Patient 1.

The accident was mainly caused by poor occupational health management of chemical substances, and the following five adjustments should be considered:

1. A special occupational health management department should be set up. At the time of the incident, there were only two persons in charge and did not have relevant knowledge and experience in occupational health.
2. The accident happened at 4 am, when the workers felt sleepy and the place was dimly lit, which are unsuitable conditions for high-risk work. This is a warning that we need to rationalize the working time of various positions.
3. The factory should regularly monitor the concentration of poisons in the air and working equipment, maintain equipment regularly, and keep the air in the working environment fresh and dry. The work area should be equipped with showers and eye washers, and emergency supplies such as 3–5% sodium bicarbonate solution should be stored.
4. The factory should equip workers with personal protective equipment, such as chemical protective clothing, goggles, and gas masks, as well as ventilation and dust removal equipment. Of note, this factory has regular medical examinations for employees.
5. The three patients only wore N95 masks at work, with no additional self-protection, and did not change their clothes after exposure, resulting in continuous skin contact with poison and increased absorption, which may be one of the reasons for the rapid progression of patient 1’s disease. Factories should deliberately provide health and safety education, emphasizing the importance of self-protection, standardized operating procedures, and dealing with toxic substances and self-rescue.

## Data availability statement

The original contributions presented in the study are included in the article/supplementary material, further inquiries can be directed to the corresponding author.

## Ethics statement

The studies involving human participants were reviewed and approved by Shandong University Qilu Hospital Ethics Committee. The patients/participants provided their written informed consent to participate in this study. Written informed consent was obtained from the individual(s) for the publication of any potentially identifiable images or data included in this article.

## Author contributions

LG, XJ, and LD obtained research funding and investigated the description of the incident. LG conceived the study and drafted the manuscript. XZ, ZZ, and MS supervised data collection. LG, XJ, and LD take responsibility for the paper as a whole. All authors contributed to the article and approved the submitted version.

## Conflict of interest

The authors declare that the research was conducted in the absence of any commercial or financial relationships that could be construed as a potential conflict of interest.

## Publisher’s note

All claims expressed in this article are solely those of the authors and do not necessarily represent those of their affiliated organizations, or those of the publisher, the editors and the reviewers. Any product that may be evaluated in this article, or claim that may be made by its manufacturer, is not guaranteed or endorsed by the publisher.
